# Annotations

**Published:** 1900-08-18

**Authors:** 


### ANNOTATIONS.

### Feeding the Navy.

Among the curiosities of life at sea are the arrangements for the meals of the sailors on board Her Majesty's ships. Early in the morning the sailors get what one may call an official feed, again at noon, and finally another at half-past four p.m. From the half-past four o'clock tea-meal till early morn all is over, and anything which the "handy man" may wish for or require he must provide for himself from the canteen out of his "savings"—that is, the money that he receives in lieu of such portions of his ration as he does not choose to draw. Now, in old days, when much of a sailor's work had to be done in spurts at odd moments, and when Jack Tar was constantly oscillating between times of great exertion and of absolute idleness, there might be much to be said for allowing a very considerable proportion of the diet to be taken at any time when opportunity might occur. A modern ship of war, however, is like a mill full of machinery, and things go on with much more regularity. Moreover, so far as the stokers are concerned, the work is so laborious, and for the time so continuous, that it is almost essential that the hours of labour and of food should, in ordinary circumstances, have some constant relation to each other, and we are glad to learn that the question of the meal hours and of the naval ration is under consideration by a departmental committee.

### The Joys of a Congressiste.

We last week took occasion to criticise the arrangements made for the opening meeting at the International Medical Congress in Paris, where, from the excessive size of the room, the whole proceedings partook of the nature of dumb show; and now for the consolation of those who were not able to go there we would, if words were possible, describe the only public entertainment given to the Congressistes, the great fête at the Luxembourg Palace, where the error took the opposite direction, the company being far too great for the rooms. But words fail. The crush, the crowd, the utter and absolute lack of all organisation, were such as can hardly be imagined, and they led to a condition of affairs which only by the merest chance escaped eventuating in a serious catastrophe. No doubt there were doors of exit, which would have been opened by some one or other if there had been a fire, or if a sudden panic had caused a fatal rush. Meanwhile heat and suffocation, torn dresses and bad temper, were, we suppose, not considered sufficient excuse for altering the arrangements or for diverting any of the throng from the great staircase, where fierce contests were progressing between those who were anxious to enjoy the delights suggested by the programme and the perspiring crowds which, having failed in their efforts, wished above all things to get out. To the *moqueur*, who being of good muscle and of ample height, could see without alarm, and being without wife or daughter could witness without sense of loss the destruction of fine dresses which was going on, the scene was not unamusing. That gentle ladies in delicate costumes fitted for spacious *salons* should be crushed and tumbled, and have literally to fight for their footing in such a crowd, was, of course, abominable, but one's sense of humour was tickled by the outrageous inappropriateness of the whole affair; and when one saw the torn coats, the damp and crumpled collars, and the general state of dishevelment and perspiration of men who have the reputation of being rather particular about their dress, when one remembered that all this was being suffered in the name of hospitality, and especially when one recalled that on entering into this pandemonium an official had actually looked one over to make sure that one's "get up" was quite correct, there certainly was room for a grim sort of merriment. We were glad to escape into the fresh air, but we were told the next day that when the thousands of disappointed ones had gone away, the more tenacious and persevering pleasure seekers had had a "high old time!" The affair, however, ought to be a lesson to the organisers of congresses that the issue of 10,000 invitations involves them in some slight responsibility, and that, in common parlance, you cannot get a quart into a pint pot. It was worse than a fiasco.

### Our Field of Practice.

Mr. George Eastes has lately drawn attention to a subject of some interest to medical men, namely, their number in relation to the population in different places, in other words, the field for practice offered by different parts of the country. The number of medical practitioners in England and Wales may be taken as 23,023, and, by dividing that number among the population, we find that each practitioner has, on an average, 1,393 persons—prospective or possible patients—out of whom to make a living. It is curious, however, to note what variations exist in the proportion of doctors to population in different places, varying from one to 615 in Brighton, and one to about 800 in the metropolis, to one to over 2,000 persons in Huddersfield, Norwich, Hull, and Bolton. Some places, indeed, would seem to be as much over-doctored as others are lacking in medical attendance, and it would be an interesting, although, perhaps, not very profitable study, to endeavour to discover whether this is due to the uncounted quacks being more abundant in certain towns, or to the people in some places being more fatalistic, more indifferent to the services of medical men, or perhaps less inclined to put faith in their powers. No doubt much is due to some towns being more attractive than others as places of residence, but we are inclined to think that something depends upon the habits of the people and their state of mind in regard to doctors, medical attendance, and medical science in general. Many people feel that there is something uncanny and almost shocking in not having a doctor when ill. There still remains, however, a great population who have not bent the knee to Baal, and are very sceptical in all that concerns doctors, nurses and other modern aids to recovery. A surprisingly large number of people still prefer to be let alone, and to meet their end at the time the Lord decides. We are reminded of the careful soul who, when it became a question of an operation to save her husband's life, advised him thus: "He says it'll be three guineas. I'd die like a man first. Don't have it done, lad. Stick to thi brass." Wherever this sort of feeling largely permeates a population medical practice is apt to be non-remunerative. Much has been said about improved sanitation as causing a diminution of medical work. It is quite a question. Where people fidget about their drains they fidget about their insides, and fidget pays the doctor.

